# Dietary Magnesium and Cardiovascular Disease: A Review with Emphasis in Epidemiological Studies

**DOI:** 10.3390/nu10020168

**Published:** 2018-02-01

**Authors:** Nuria Rosique-Esteban, Marta Guasch-Ferré, Pablo Hernández-Alonso, Jordi Salas-Salvadó

**Affiliations:** 1 Human Nutrition Unit, Department of Biochemistry and Biotechnology, Faculty of Medicine and Health Sciences, University Hospital of Sant Joan de Reus, Pere Virgili Institute for Health Research, Rovira i Virgili University, St/Sant Llorenç 21, 43201 Reus, Spain; nuria.rosique@urv.cat (N.R.-E.); mguasch@hsph.harvard.edu (M.G.-F.); pablo.hernandez@urv.cat (P.H.-A.); 2 CIBERobn Physiopathology of Obesity and Nutrition, Institute of Health Carlos III (ISCIII), 28029 Madrid, Spain; 3 Department of Nutrition, Harvard T.H. Chan School of Public Health, Boston, MA 02115, USA

**Keywords:** magnesium, cardiovascular, type 2 diabetes, metabolic syndrome, mortality, death, epidemiological studies, inflammation, oxidation

## Abstract

Magnesium (Mg) is an essential dietary element for humans involved in key biological processes. A growing body of evidence from epidemiological studies, randomized controlled trials (RCTs) and meta-analyses have indicated inverse associations between Mg intake and cardiovascular diseases (CVD). The present review aims to summarize recent scientific evidence on the topic, with a focus on data from epidemiological studies assessing the associations between Mg intake and major cardiovascular (CV) risk factors and CVD. We also aimed to review current literature on circulating Mg and CVD, as well as potential biological processes underlying these observations. We concluded that high Mg intake is associated with lower risk of major CV risk factors (mainly metabolic syndrome, diabetes and hypertension), stroke and total CVD. Higher levels of circulating Mg are associated with lower risk of CVD, mainly ischemic heart disease and coronary heart disease. Further, RCTs and prospective studies would help to clarify whether Mg intake and Mg circulating levels may also protect against other CVDs and CVD death.

## 1. Introduction

**Figure 1:**
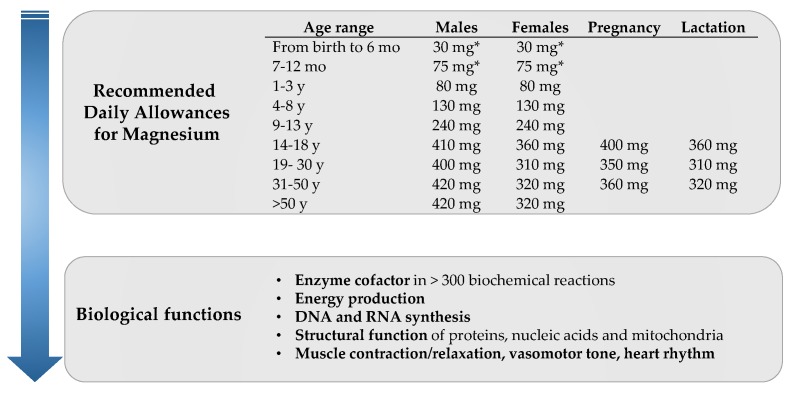
Summary of current Recommended Daily Allowances (RDAs) for magnesium intake [2] and key biological functions of magnesium. Abbreviations: mo, months; y, years. * indicates Adequate Intake.

Magnesium (Mg) is an essential mineral for human health, representing the fourth most abundant mineral in the body. It is involved in important metabolic processes including ATP-dependent biochemical reactions, synthesis of DNA, RNA expression, cell signaling at muscle and nerve levels, and glucose and blood pressure (BP) control, among others (Figure 1) [1]. To guarantee the correct functioning of these processes, humans require a continuous supply of Mg from exogenous sources, i.e., dietary intake. Nuts, seeds, legumes, whole-grain cereals, leafy vegetables or water are well-recognized dietary sources of Mg (Table 1), regular consumption of which enables reaching the recommended dietary allowance currently set in 420 mg/day for adult men and 320 mg/day for adult women [2]. Mg requirements vary across age, sex and physiological situations (Figure 1). Dietary surveys in Europe and United States have shown that daily allowance of Mg are unmet in a large proportion of the population, probably as a result of following Western dietary patterns [2]. Several publications and recent meta-analyses have revealed inverse associations of dietary Mg intake with the risk of cardiovascular disease (CVD); cardiovascular (CV) risk factors including type 2 diabetes (T2D), metabolic syndrome (MetS) or hypertension; and total mortality [3]. Similarly, chronic Mg deficiency (defined as circulating [Mg^2+^] < 1.8 mg/dL) has been associated with increased risk of several cardio-metabolic conditions [4,5]. A low inter-correlation between dietary Mg and circulating [Mg^2+^] has been described [6], possibly as a result of the tight homeostatic regulation of [Mg^2+^] through renal reabsorption and excretion, although the determinants of variation within the normal physiologic range are not well understood. For instance, genetic variations in single nucleotide polymorphisms may account for less than 2% of the variance in serum [Mg^2+^] [7], and the understanding of the influence of endocrine factors on Mg homeostasis need to be clarified [8]. Additionally, serum Mg only represents a minimal proportion of the Mg present in the entire body and thus intracellular [Mg^2+^] may be a more accurate method reflecting Mg status yet with additional difficulties to be measured [9]. Despite of this, and their low inter-correlation, both Mg intake and circulating [Mg^2+^] have been repeatedly associated with CV health [3,4,5], and therefore both are of great research interest.

The present review aims to summarize the most up-to-date scientific evidence on dietary Mg intake and circulating Mg, in relation to CVD. To this end, efforts have been made to describe most recent meta-analyses of prospective studies and RCTs on the topic, as well as current literature on Mg intake and CV risk factors. Recent investigations relating circulating Mg and CVD and plausible biological mechanisms underlying the potential beneficial role of dietary Mg intake on CV health have been reviewed. Because the present review is not based on a systematic search, some articles may not have been identified and publication bias should be acknowledged. However, all the authors independently conducted the literature search.

## 2. Dietary Magnesium and Cardiovascular Disease Risk Factors

Experimental and observational studies have shown that higher Mg intake can exert beneficial effects on CV risk factors by improving glucose and insulin metabolism, enhancing endothelium-dependent vasodilation, ameliorating lipid profile and by its actions as an antihypertensive and anti-inflammatory agent [11].

### 2.1. Type 2 Diabetes and Metabolic Syndrome

Mg plays an important role in glucose and insulin metabolism, likely via insulin resistance (IR) pathways and directly affecting glucose transporter protein activity 4 (GLUT 4) [12]. A large body of observational literature has suggested that higher Mg intake is associated with a lower risk of T2D and MetS. Indeed, several meta-analyses on this topic have already been conducted. The most recent one included 637,922 individuals with 26,828 T2D cases from twenty-five cohort studies. Results of this meta-analysis indicated that compared with those participants in the lowest Mg consumption category, the risk of T2D was reduced by 17% across all the studies; 19% in women and 16% in men. In addition, a linear dose–response relationship was observed between Mg intake and T2D, such that risk was 8%–13% lower per 100 mg/day increment in intake [13]. Previous meta-analyses evaluating Mg intake and T2D have also found consistent inverse associations [14,15]. More recently, a large prospective cohort study including more than 200,000 participants followed for over 28 years from the Nurses’ Health Study (NHS) I, NHS II and Health Professionals’ Follow-up study (HPFS) showed that, in pooled analysis across the three cohorts, those with the highest Mg intake (intake ranging from 350 to 500 mg/day) had 15% lower risk of T2D compared to those in the lowest Mg intake group (Hazard Ratio (HR) in the highest vs. the lowest quintile: 0.85 (95% CI 0.80–0.91)) [16]. This evidence has been further confirmed by clinical trials on Mg supplementation indicating beneficial effects of Mg on markers of glucose and insulin metabolism in individuals with and without T2D [17,18,19]. Findings from a recent meta-analysis of RCTs on the effects of supplemental Mg have demonstrated a significant effect of Mg on the Homeostatic model assessment of insulin resistance (HOMA-IR) index (weighted mean difference (WMD): −0.67, 95% CI: −1.20, −0.14); however, reductions in IR and fasting glucose were only observed when trials had a follow-up larger than four months [17].

IR is indeed one of the underlying causes of a cluster of risk factors for CVD, namely MetS. Evidence exists suggesting that there are potential benefits of dietary Mg in preventing MetS and its components [20,21]. A recent meta-analysis on this topic has summarized the evidence of nine observational studies including 31,876 participants [22]. Results indicated that higher consumption of Mg was associated with a lower risk of MetS (Odds Ratio (OR): 0.73, 95% CI, (0.62–0.86)) compared to participants in the lowest categories of Mg consumption. However, this meta-analysis combined the estimates from cross-sectional and prospective cohort studies altogether, and the association between components of the MetS and Mg intake was not assessed because it was not reported in the majority of the included studies [22]. However, for example, in a study conducted in a sample of 535 participants, Mg intake was inversely associated not only with the MetS but significant inverse relationships were observed between Mg intake, and body mass index (BMI) (OR: 0.47, 95% CI: 0.22–1.00, *p* for trend = 0.03), and fasting glucose (OR: 0.41, 95% CI 0.22–0.77, *p* for trend = 0.005) [23]. Two other review articles published in 2014 also summarized the associations between Mg intake and MetS in several studies [24,25]. Ju et al. [24] found an inverse dose–response association when pooling the data of nine observational studies, and Dibaba et al. [25] also observed consistent inverse associations between Mg intake and MetS when combining the data of six cross-sectional studies.

Overall, data from observational studies suggest a beneficial role of Mg intake in T2D prevention, whereas results from intervention studies have shown beneficial effects on HOMA-IR and fasting glucose. Given that these are surrogate biomarkers, further RCTs of Mg intake should focus on evaluating major endpoints, such as T2D incidence. Findings from studies on dietary Mg and MetS point to an inverse association with the prevalence of MetS, but further larger and long-term prospective studies and RCTs are needed to elucidate the role of Mg intake on MetS and its components.

### 2.2. Hypertension and Endothelial Function

High blood pressure or hypertension has been established as a strong risk factor for CVD. Among others, a diet rich in Na^+^ and an inadequate dietary intake of other minerals including K^+^, Ca^+^ and Mg has been linked to hypertension [26]. Previous evidence has indicated that Mg deficiency might affect BP, thus leading to hypertension, and oral Mg supplementation may act as a mild antihypertensive agent [27]. In a recent meta-analysis on this topic, the authors have pooled the estimates on dietary Mg and hypertension of six prospective cohort studies including 20,119 cases and 180,566 participants [13]. The summary estimate indicated a statistically significant inverse association between dietary Mg and hypertension risk (pooled RR comparing extreme categories = 0.92; 95% CI: 0.86, 0.98) without apparent evidence of heterogeneity between studies. The range of dietary Mg intake among the included studies was 96–425 mg/day, and the follow-up ranged from 4 to 15 years. In addition, a 100 mg/day increment in Mg intake was associated with a 5% reduction in the risk of hypertension (RR = 0.95; 95% CI: 0.90, 1.00) [13]. These findings were consistent with those from RCTs examining the effect of Mg supplementation on BP. The most recent meta-analysis has identified eleven RCTs including 543 participants with preclinical or non-communicable diseases who were followed for a range of 1–6 months [28]. The weighted overall effects indicated that the group who was supplemented with oral Mg had a significantly greater reduction in both systolic BP (Standardized Mean Difference (SMD): −0.20; 95% CI: −0.37, −0.03) and diastolic BP (SMD: −0.27; 95% CI: −0.52, −0.03) than did the control group. Mg supplementation resulted in a mean reduction of 4.18 mmHg in systolic BP and 2.27 mmHg in diastolic BP [28]. Previous meta-analysis and systematic reviews on dietary Mg, BP and hypertension have also suggested potential benefits of dietary Mg [29,30], yet showing a more moderate effect, possibly as a result of combining individuals with and without chronic diseases in the pooled analysis.

Hypertension is indeed an important risk factor for endothelial dysfunction, and an essential step in the pathogenesis of atherosclerosis that ultimately may lead to coronary artery disease (CHD). Mg is involved in several essential physiological biochemical and cellular processes regulating CV function; in addition, it plays a crucial role in modulating vascular smooth muscle tone and endothelial function [31]. Several observational studies and clinical trials have evaluated the relationship between dietary Mg and biomarkers of endothelial function. As an illustration, in a cross-sectional study of 657 women of the NHS, in the age-adjusted linear regression analyses, Mg intake was inversely associated with plasma concentrations of E-selectin (*p* = 0.001), and soluble intercellular adhesion molecule 1 (sICAM-1) (*p* = 0.03). After further adjustment for physical activity, smoking status, alcohol use, postmenopausal hormone use, and BMI, dietary Mg intake remained inversely associated with E-selectin [32]. In another report of the Women’s Health Initiative Observational Study including 3713 postmenopausal women, dietary Mg intake was inversely associated with plasma concentrations of soluble vascular cell adhesion molecule 1 (sVCAM-1) and E-selectin, independent of known risk factors for metabolic outcomes. Specifically, an increase of 100 mg/day Mg was inversely associated with sVCAM-1 (−0.04 ± 0.02 ng/mL; *p* = 0.07) [33]. Finally, a randomized, double-blind, placebo-controlled trial has indicated that oral Mg supplementation (30 mmol Mg/day) for six months resulted in a significant improvement in endothelium-dependent brachial artery flow-mediated vasodilation in 50 patients with coronary artery disease, indicating a beneficial effect of Mg on endothelial function [34].

In summary, several lines of evidence have suggested a potential beneficial link between high dietary Mg and low blood pressure, particularly among those individuals with either preclinical or non-communicable diseases. Furthermore, Mg intake seems to improve endothelial function but more studies are needed to confirm these associations in the long-term.

### 2.3. Lipid Profile

Dyslipidemia, one of the components of MetS, is a modifiable risk factor for the development of atherosclerosis and CVD [35]. Besides the potential benefits of dietary and supplemental Mg on IR, BP and endothelial function, several studies have also indicated that dietary Mg may be linked to an improvement in lipid profile including a decrease in low-density lipoprotein (LDL) cholesterol and triglycerides levels and an increase in high-density lipoprotein (HDL) cholesterol [36,37]. In a cross-sectional study, Guerrero-Romero et al. compared 192 individuals with MetS with 384 healthy age- and sex-matched controls. Of all of the MetS components, hypomagnesemia was most closely related with dyslipidemia (OR: 2.8; 95% CI: 1.3, 2.9) and hypertension (OR: 1.9; 95% CI: 1.4, 2.8) [38]. Similarly, in a cross-sectional analysis of the Tehran Lipid and Glucose Study including 2504 participants which evaluated the associations between dietary Mg and MetS and its components, Mg intake (mean intake of 349 mg/day) was inversely associated with triglycerides (β = −0.058, *P* = 0.009) [39]. In another recent cross-sectional study including 4443 individuals from the European Prospective Investigation into Cancer (EPIC)-Norfolk cohort, higher Mg intake was inversely associated with total cholesterol (*p* for trend = 0.02 men and 0.04 women) [40]. However, the evidence from longitudinal observational studies on dietary Mg and lipid profile biomarkers is limited. Most of the evidence regarding Mg and blood lipids comes from RCTs on oral Mg supplementation. A recent meta-analysis of RCTs did not show significant effects of Mg on plasma concentrations of total cholesterol, LDL-cholesterol, HDL-cholesterol and triglycerides [41]. The authors suggested that this may be explained by the inter-study heterogeneity due to the different formulations and salts of Mg used across the studies and the heterogeneity of the studied populations [41]. Nonetheless, the authors reported a significant reduction on LDL-cholesterol and triglyceride concentrations after Mg supplementation in a subgroup of studies in participants with hypercholesterolemia and hypertriglyceridemia, respectively. Thus, results suggested that according to the metabolic status, Mg may affect the lipid profile [41]. Moreover, in a randomized trial were 214 participants were administered a Mg-rich diet (1142 ± 225 mg/day) and 216 participants followed their usual diet (438 ± 118 mg/day) for 12 weeks, there was a significant decrease in total serum cholesterol (10.7%), LDL-cholesterol (10.5%) and triglycerides (10.1%) in participants following a Mg-rich diet compared to the baseline concentrations; no such changes were evident in participants following a usual diet. HDL-cholesterol showed a marginal mean decrease of 0.8 mg/dL in the control group and 2.0 mg/dL increase in the intervention group [36].

In conclusion, evidence from RCTs have suggested plausible beneficial effects of oral Mg supplementation for improving some lipid parameters, but improvements were only evident in individuals with dyslipidemia. Therefore, longer and larger trials and prospective cohort studies on this topic are still required.

## 3. Dietary Magnesium and Cardiovascular Disease

Most of the scientific literature on dietary Mg intake and CVD derive from prospective studies. Recently, an increasing number of meta-analyses have summarized previous literature on this topic, thus providing a more concise and public health-directed overview. The present section focuses on the evidence from epidemiological studies in relation with dietary Mg intake and major CVD events, as well as with CV death.

### 3.1. Stroke

Current scientific evidence on dietary Mg and the risk of stroke is mostly available from prospective cohort studies in North American, European and, to a lesser degree, from Asian populations. Previous and more recent meta-analyses have exemplified the available data [3,42,43,44], reporting similar results on the dose-dependent protective effect of Mg intake on stroke risk. While previous meta-analyses have reported between 2% and 13% protection against total stroke for an increment of 100 mg/day intake of dietary Mg [42,43,44], Fang et al. have confirmed this protective effect in the most updated analysis conducted in 2016, including fourteen prospective cohort studies [3]. In this updated meta-analysis, the authors have also reported 22% lower risk of stroke (RR: 0.88; 95% CI, 0.82, 0.95) in those individuals in the highest vs. the lowest categories of dietary Mg intake, which is in agreement with findings reported in previous studies [43]. It is important to highlight that these meta-analyses have shown null to low heterogeneity between studies, they have found no evidence of publication bias and they included studies adjusting for several potential confounders. Taken together, current evidence from prospective studies in large populations across the world indicated a dose-dependent inverse association between dietary Mg and stroke incidence.

### 3.2. Coronary Heart Disease

Coronary Heart Disease (CHD) in relation with dietary Mg intake has been extensively investigated in large population cohorts in America and Asia, including the NHS, the HPFS and the Shanghai Women’s and Men’s Healthy study, among other cohorts [6,45,46,47,48,49]. However, most of these studies did not show a significant association when comparing the highest vs. the lowest category of Mg intake in relation to the risk of CHD in either men or women. For instance, in an analysis of more than 86,000 healthy American nurses, no significant associations were found comparing the highest vs. the lowest quintile of Mg intake on total and non-fatal CHD over a median of 28 years of follow-up [6]. In this study, and similar to other analysis in the same cohort [47], Mg intake included those from food sources and supplements, whereas the rest of the investigations only included Mg intake from foods [45,46,48]. A pooled analysis by Fang and collaborators [3] including nine different prospective cohorts have revealed inverse borderline associations with the highest category of Mg intake on CHD risk (RR: 0.90; 95% CI, 0.80, 0.99), with low heterogeneity across studies. In an additional dose–response analysis, the authors reported non-significant associations for higher Mg intake on CHD, with low to medium heterogeneity among studies. On the contrary, a recently published prospective study conducted in a large cohort of community-based Japanese adults [49] with a total of 1283 cases of CHD has revealed a protection against CHD in men in the fourth (HR: 0.70 95% CI, 0.50, 0.99) and fifth quintile (HR: 0.66 95% CI, 0.44, 0.97) of Mg intake compared to the lowest quintile (*P* for linear trend = 0.036). Overall, current evidence on Mg intake and CHD suggests a non-significant inverse association in most of the populations studied. Nevertheless, additional prospective studies and clinical trials on this topic should focus on addressing plausible age, sex and country differences.

### 3.3. Heart Failure

Epidemiological evidence on the associations between dietary Mg and heart failure (HF) are currently limited to two independent American and Japanese cohort studies [50,51]. Although large risk reductions in HF incidence have been reported in the highest vs. lowest Mg intake among black American individuals and Japanese women, the associations were non-significant for Japanese men [51]. These differences may be due to the lower Mg intake in Japanese men compared to their American counterparts (294 and 474 mg/day, respectively), or due to biological differences between the study populations. Nevertheless, a recently published meta-analysis [3] including these two independent cohorts has shown a strong inverse association with HF for the highest vs. the lowest categories (RR: 0.69; 95% CI, 0.52, 0.91) and also per an increment of 100 mg/day of Mg intake (RR: 0.78, 95% CI, 0.69, 0.89), both with no apparent heterogeneity. Further data from prospective studies and clinical trials in other countries and ethnicity-specific populations is required to have an extensive insight on the possible role of Mg in preventing HF.

### 3.4. Atrial Fibrillation

The associations between dietary Mg intake and atrial fibrillation, a common cardiac arrhythmia, have barely been explored in epidemiological and experimental settings. To the best of our knowledge, only one prospective cohort study has, so far, addressed this topic [52]. The authors followed more than 14,000 middle-aged American whites and African American participants from the ARIC study for 20.6 years, reporting 1755 incident cases of atrial fibrillation. Compared to the middle quintile (median dietary Mg: 223.2–264.8 mg/day), those individuals in the highest (median dietary Mg: ≥320.1 mg/day) or the lowest quintile of Mg intake (median dietary Mg < 180.9 mg/day)—exclusively from dietary sources, as assessed by food frequency questionnaire—did not show significantly higher or lower incidence of atrial fibrillation. These results were found regardless of sex or ethnicity, as the authors reported no significant interactions with dietary Mg. Despite these results, previous experimental studies under controlled conditions in individuals with similar characteristics have demonstrated that dietary-induced Mg depletion can induce heart rhythm changes with few participants showing atrial flutter and fibrillation [53], and that subsequent Mg repletion with supplements reverse these heart rhythm changes. These limited and mixed results illustrate the need of more epidemiological and experimental evidences to elucidate the link between dietary Mg intake and atrial fibrillation across different populations.

### 3.5. Cardiovascular Death

Several epidemiological studies across North American [6,47,54,55,56], Asian [48,51], and, to a lesser degree, European populations [57] of middle-aged men and women have prospectively evaluated the associations between dietary Mg intake and risk of CV death yielding inconclusive results. With the purpose of summarizing the existing evidence on the topic, two meta-analyses have been published to date [58,59]. In a first meta-analysis, Xu et al. [58] included a total of six prospective studies comprising more than 200,000 men and women with a follow-up ranging between 10 and 26 years. In the pooled analysis, those participants in highest category of Mg intake comparted to those in the lowest category, showed no significant differences regarding total CV death risk, yet high heterogeneity across studies was detected. However, further inspection of subgroup analyses revealed a 29% protection only in women (RR: 0.71; 95% CI, 0.60, 0.84).

In a more recent updated meta-analysis [59] including more than 400,000 adults from different cohorts who were followed for 5 to 28 years, the summary estimate comparing individuals at the higher vs. the lowest categories of dietary Mg intake showed a protection of 14% (HR: 0.86; 95% CI, 0.81, 0.91) against the risk of CV death, with high heterogeneity among studies and no evidence of publication bias. Subgroup analyses revealed that this protection corresponded to 16% in women and 8% in men. Further inspection of the subtypes of CVD death showed that dietary Mg intake was inversely and significantly associated with lower risk for CHD, heart failure and sudden cardiac death. Additional dose–response analysis showed a protection of 25% (HR: 0.75; 95% CI, 0.58, 0.99) in women for the increment of 100 mg/day of Mg intake.

Overall, current evidence in relation to dietary Mg intake and death for total CVD and CV subtypes shows a protective role, particularly in women. Nevertheless, the results derived from meta-analyses should be interpreted with caution given the high heterogeneity found across the studies. Further RCTs on dietary Mg intake should focus on assessing CV hard endpoints to clarify current available evidence aroused from prospective studies.

## 4. Circulating Magnesium and Cardiovascular Disease

Several prospective studies have analyzed the associations between peripheral levels of Mg or urine Mg excretion (Mg status) and the risk of several CVD including cardiac arrhythmias, congestive HF, CHD, stroke, sudden death and death from all these causes.

Although a poor correlation between dietary Mg and plasma levels of this mineral has been reported in several prospective studies [6,45], evidence exists demonstrating that very low Mg diets may lead to low serum Mg levels, and a recent meta-analysis of RCTs demonstrated significant dose and time responses of circulating [Mg^2+^] and 24 h urine Mg excretion to oral Mg supplementation [60].

Therefore, in this section, we summarize the epidemiological evidence in the literature analyzing the association between low circulating (serum/plasma) [Mg^2+^] or urinary Mg excretion and CVDs.

### 4.1. Cardiovascular Disease, Coronary Disease and Stroke

The most recent systematic review and meta-analysis analyzing the associations between peripheral [Mg^2+^] and CVD identified significant associations of circulating Mg and risk of CVD events [4]. Circulating [Mg^2+^] (per 0.2 mmol/L increment) was associated with a 30% lower risk of CVD, with trends towards a lower risk of ischemic heart disease (IHD) and fatal IHD. This meta-analysis, including a total of 313,041 individuals and documenting 4106 CVD, 3215 IHD, and 1528 fatal IHD events, provides the most robust evidence to date of the associations between circulating [Mg^2+^] across their usual physiologic ranges with CVD risk. However, a moderate heterogeneity between the studies analyzed was reported.

Since then, two additional large prospective studies examining this relation have been conducted. Chiuve et al. [6] conducted a nested case–control analysis in the context of the NHS with 458 cases of incident CHD matched to controls. Higher plasma [Mg^2+^] was associated in an L-shaped fashion with lower risk of CHD, although this association was not independent of CVD biomarkers. In contrast, in the Prevention of Renal and Vascular End-Stage Disease (PREVEND) study—a prospective population-based cohort study—low urinary Mg excretion was independently associated with a higher risk of IHD incidence. Nevertheless, no significant associations were observed between plasma levels of this mineral and IHD incidence or death [61].

In relation to stroke, several cross-sectional and retrospective case–control studies have reported lower serum [Mg^2+^] in those individuals with acute stroke compared with healthy controls. However, in these studies Mg was not measured before stroke diagnosis and thus hypomagnesemia may have been a consequence rather than a cause of stroke in these patients. No prospective association between serum [Mg^2+^] and risk of stroke was reported in the ARIC Study cohort after adjusting by several confounders (based on 577 ischemic stroke cases in men and women with 16 years of follow-up) [62]. In the NHS, plasma Mg levels were not associated with the risk of ischemic stroke in women [63]. However, women with [Mg^2+^] levels < 0.82 mmol/L had a 57% higher risk of ischemic stroke, and this association remained unchanged after controlling for other factors associated with Mg levels and stroke risk.

### 4.2. Atrial Fibrillation and Sudden Death

Low serum [Mg^2+^] has been linked to an increased risk of atrial fibrillation (AF) after cardiac surgery. However, it is unknown whether hypomagnesemia predisposes to AF in the general population. Two prospective studies have analyzed the association between serum [Mg^2+^] and AF risk in healthy populations and in individuals at risk of CVD. In the context of the Framingham Health Study, low serum [Mg^2+^] was moderately associated with the development of AF in individuals without previous history of CVD [64]. In the ARIC cohort, an L-shaped association between serum [Mg^2+^] and incident AF was also identified, with the highest risk of AF in those individuals with low serum [Mg^2+^], and a lower risk at more normal and elevated [Mg^2+^] levels [52]. This association, not different among white and African populations, was evident even after adjustment for the most important recognized risk factors of AF.

### 4.3. Left Ventricular Hypertrophy and Heart Failure

A few lines of evidence suggest that low circulating [Mg^2+^] may predict left ventricular hypertrophy or HF among population-based individuals. For example, low serum [Mg^2+^] has been demonstrated to predict higher increase in left ventricular mass over five years of follow-up in the population-based longitudinal Study of Health in Pomerania including 1348 individuals with echocardiographic data [65]. This prognostic impact was regardless of sex, age and traditional CV risk factors including prevalent hypertension. In relation to cardiac insufficiency, in the ARIC Study, a North American population-based cohort, low serum [Mg^2+^] and high serum phosphorus and calcium concentrations were independently associated with greater risk of incident HF after controlling for several potential confounders, with the association remaining consistent across sex and ethnicity [66]. In a population-based prospective study of middle-aged Finnish men without HF at baseline, the Kuopio Ischemic Heart Disease Study, a decreased risk for incident HF with increasing serum [Mg^2+^] was also reported [67]. This association persisted after controlling for baseline characteristics, predictors of incident HF, metabolic and renal biomarkers, and other related micronutrients.

### 4.4. Atherosclerosis and Coronary Artery Calcification

In few epidemiological studies, low circulating [Mg^2+^] was inversely associated with atherosclerosis and coronary artery calcification. For example, in the ARIC study, decreased serum [Mg^2+^] were associated with increased mean carotid wall thickness in women [68]. Hashimoto et al. [69] analyzed 728 subjects from the general Japanese population and also found that serum [Mg^2+^] to be inversely associated with intima-media thickness of the common carotid artery, and with the presence of atherosclerotic plaque. In two recent cross-sectional studies conducted in Korean and Mexican populations free from CVD, low serum [Mg^2+^] were also associated with coronary artery calcification [70,71].

### 4.5. Cardiovascular Death

In a systematic review and meta-analysis, using the results of four studies including 27,293 individuals and 1528 cases the association between circulating [Mg^2+^] and fatal IHD risk has been evaluated [4], showing a trend towards a lower risk in those individuals with lower [Mg^2+^], with substantial between-study heterogeneity. In secondary analyses using fixed effects, circulating [Mg^2+^] were associated with a significantly lower risk of fatal IHD. Nevertheless, meta-regression analysis did not show statistically significant sources of heterogeneity, although statistical power to identify heterogeneity was limited because only four studies were included in the analysis.

Peripheral [Mg^2+^] were also inversely associated with fatal CHD in the NHS [6], and with fatal stroke in the NHANES I Epidemiologic Follow-up Study [72]. Low serum [Mg^2+^] were also associated with an increased risk of CHD death and sudden cardiac death in a recent European prospective population-based cohort study [73].

Finally, in a recent systematic review and meta-analysis of studies conducted in HF patients, hypermagnesemia with serum [Mg^2+^] ≥1.05 mmol/L was associated with an increased risk of CVD death and all-cause death, but this was not observed for hypomagnesemia [74]. However, these findings were limited to elderly patients with chronic HF who had reduced left ventricular systolic function.

## 5. Plausible Mechanisms Connecting Magnesium and Cardiovascular Disease

**Figure 2:**
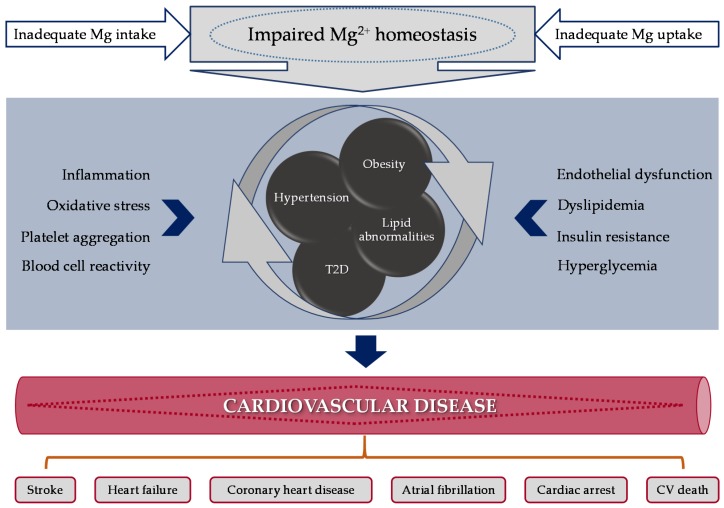
Mechanisms linking magnesium abnormalities (intake or circulating levels) with molecular outcomes leading to CV risk factors that may induce CV disease. Abbreviations: CV, cardiovascular; Mg, magnesium; T2D, type 2 diabetes.

The inverse association between Mg intake and IR, hyperglycemia, dyslipidemia, hypertension, and markers of inflammation may justify the protective effect of dietary Mg on CVD. As reviewed in previous sections, several RCTs and epidemiological studies have widely indicated that higher dietary Mg intake and/or circulating [Mg^2+^] are associated with lower risk of CVD, such as IHD and sudden cardiac death. Given the participation of Mg^2+^ in a wide range of biological pathways and outcomes, it is not surprising that alterations in Mg homeostasis may influence different disease status, such as T2D and hypertension, together with CV events (Figure 2). Numerous studies have reported an increased oxidative stress during Mg-deficiency (Mg-D), including enhanced erythrocyte, tissue, and lipoprotein peroxidation, implicated in the early stages and progression of CVDs [75,76,77,78,79].

In this paper, we have mainly focused in outcomes related to CVD and/or their risk factors. Studies investigating these mechanisms have mostly focused on the effects of Mg supplementation or circulating [Mg^2+^] levels on inflammation and/or oxidative processes. However, specific in vitro and/or in vivo research has also been conducted to explore the overall processes affecting CV outcomes or CV risk factors (Figure 2).

### 5.1. Oxidative and Inflammatory Stress

Already at the beginning of the 20th century, after the discovery of Mg as an essential nutrient, Mg-D was linked to inflammation [80]. It is well-characterized that a previous phase of Mg depletion renders cells—particularly myocardial tissue—more sensitive to oxidative stress [75,76,77]. However, supplementation with Mg led to an increase in antioxidant blood concentrations [75,81,82]. It has been hypothesized either that Mg-D induces oxidative stress due to its pro-inflammatory effect, or that Mg itself possess antioxidant properties, scavenging oxygen radicals [75,76]. Mg-D may also induce a pro-inflammatory response by modulation of the intracellular Ca^2+^ concentration, release of neurotransmitters, or the activation of nuclear factor-kB implicated in the regulation of immune and inflammatory responses [76].

Freedman and collaborators demonstrated during the nineties the participation of free radicals in Mg-D cardiomyopathy and the incapacity of Mg-deficient animals to withstand an in vivo oxidative stress [78,79]. They evaluated the effect on Syrian hamsters being fed for 14 days with a Mg-deficient diet or a Mg-supplemented control diet, showing that Mg-D increases the susceptibility of the CV system to oxidative stress [79]. However, researchers also found that the concentration of nitric oxide (NO) is markedly increased in plasma of Mg-deficient rats, thus providing an additional cause of oxidative lesions through formation of peroxynitrite [83].

In vitro studies were also performed and the effect of Mg-deficient culture on endothelial cell susceptibility to oxidative stress was examined. Bovine endothelial cells were cultured in either control sufficient (0.8 mM) or deficient (0.4 mM) levels of MgCl_2_. Results derived from this investigation suggest that in vitro Mg-D enhances free radical-induced intracellular oxidation and cytotoxicity in endothelial cells [84]. To further explore these effects, Wiles et al. examined the exposure of acute Mg-D to this cell type. Decreasing [Mg^2+^] (≤0.25 mM) significantly increased endothelial cell oxidant production relative to control [Mg^2+^] (1 mM). This suggested that acute Mg-D is sufficient for the induction of endothelial cells oxidant production, the extent of which may determine, at least in part, the extent of endothelial cells dysfunction/injury associated with chronic Mg-D [85].

Severe dietary Mg restriction (9% of the recommended dietary allowance (RDA) is sufficient to induce a pro-inflammatory neurogenic response in rats [86]. Thus, Kramer and collaborators evaluated in 2003 whether less severe dietary Mg restriction modulates the extent of both the neurogenic/oxidative responses in vivo and ischemia/reperfusion injury in vitro. Male Sprague-Dawley rats maintained on Mg (40% RDA) or Mg (100% RDA) diets during the first three weeks were compared with the 9% RDA Mg group. They found that erythrocyte glutathione and plasma malondialdehyde levels revealed a direct relationship between the severity of oxidative stress and hypomagnesemia. Thus, suggesting that varying dietary Mg intake directly influences the magnitude of the neurogenic/oxidative responses in vivo and the resultant myocardial tolerance to ischemia/reperfusion stress [87]. Because it has been demonstrated that Mg-D promotes inflammation in vivo, Bernardini and collaborators evaluated the effect of different [Mg^2+^] on microvascular 1G11 cells [88]. They reported that low [Mg^2+^] inhibits endothelial growth and migration, while it increases some inflammatory- and endothelial-related markers (IL-1a, IL-6, NO and VCAM), whereas high [Mg^2+^] stimulates cell proliferation and migration. This result demonstrated a direct role of Mg in the modulation of microvascular functions and provided a molecular explanation to the link among Mg, angiogenesis and inflammation observed in in vivo CV models [88].

### 5.2. Lipid Profile and Peroxidation

Rayssiguier and collaborators demonstrated for the first time in 1993, using Wistar rats, that dietary Mg-D affects susceptibility of lipoproteins and tissues to peroxidation [89]. The destruction of membrane lipids and the end-products of such lipid peroxidation reactions are especially dangerous for the viability of cells and tissues, hence comprising a crucial step in the pathogenesis of several CVD [90]. Several years later, Morrill et al. monitored changes in rat lipid extracts of aortic and cerebrovascular smooth muscle as extracellular [Mg^2+^] was being reduced. They found, within the pathophysiological range of Mg^2+^, a progressive reduction in fatty acid chain length and double bond content—which results in fatty acid truncation—as [Mg^2+^] is lowered. A decrease in lipid oxidation in the presence of elevated [Mg^2+^] could contribute to the apparent protective role of increased Mg intake on vascular function in humans [91].

Postprandial accumulation of triglyceride-rich lipoproteins (TGRLP) is an important characteristic of hyperlipidemia associated with Mg-D in animal models. Control and Mg-deficient rats were fed for eight days on adequate or Mg-D diets. Researchers tested the susceptibility of TGRLP isolated from control and treated rats to cell-dependent peroxidation and the effect of these lipoproteins on in vitro cultured vascular smooth muscle cells (VSMC). Results showed a higher oxidation in the case of Mg-deficient rats. This outcome warrants the atherogenic properties of Mg-D, which is accompanied by hyperlipidemia and which affects two important linked pathways: lipoprotein peroxidation and VSMC proliferation [92]. Moreover, additional results proved that TGRLP-oxidative damage is not the result of a decrease in vitamin E antioxidant status [93].

Altura and collaborators investigated in vivo the etiology of cardiac diseases. They examined the effects of Mg depletion on myocardial bioenergetic, carbohydrate, lipid and phospholipid metabolism. Rat myocardial biopsies were studied after being fed for 3 months with a dietary Mg restriction (20% normal dietary intake). Dietary Mg-D resulted in a drop in myocardial glycogen, glucose 6-phosphate, glycerol phosphate, as well as the contents of different phospholipids, illustrating impaired phospholipid metabolism and mitochondrial oxidation of long-chain fatty acids. These observations are consistent with the principle that prolonged low [Mg^2+^] can result in marked reduction in O_2_ and substrate delivery to the cardiac myocytes, with concomitant changes in membrane phospholipids (potentially resulting in a pro-oxidant state) probably as a result of coronary vasoconstriction [94].

### 5.3. Glucose Homeostasis/Type 2 Diabetes

The means whereby hypomagnesemia may promote or worsen existing T2D have not been fully unraveled. It has been suggested that Mg^2+^ regulates cellular glucose metabolism directly as it serves as an important cofactor for various enzymes and acts as a second messenger for insulin [95]. Importantly, it was also observed that insulin enhances intracellular Mg^2+^ uptake and this in turn mediates diverse effects ascribed to insulin [95]. Furthermore, low [Mg^2+^] may induce altered cellular glucose transport, reduced pancreatic insulin secretion, defective post-receptor insulin signaling and/or altered insulin–insulin receptor interactions [96] and thus aggravate the processes related to IR, important risk factor for CVD [95].

### 5.4. Endothelial Function, Blood Pressure and Hypertension

In spite of considerable research, the exact underlying causes for altered Mg metabolism in hypertensive individuals remain unclear [97]. It is assumed that inadequate dietary Mg intake or a malfunction on its metabolism can lead to vasospasm and endothelial damage [98]. Therefore, as it has already introduced before, Mg-D might affect BP and/or endothelial function, which may promote hypertension.

In an in vitro study, Maier et al. cultured human umbilical vein endothelial cells for various times in media containing different [Mg^2+^] (2–10 mM) and compared them to the corresponding controls (1 mM). High [Mg^2+^] stimulated endothelial proliferation and enhanced the mitogenic response to angiogenic factors. In addition, high [Mg^2+^] did not modulate the levels of plasminogen activator inhibitor-1, but enhanced the synthesis of NO, in part through the up-regulation of endothelial nitric oxide synthase. Thus, as it induces the synthesis of NO, Mg supplementation could be a helpful management approach in hypertension as well as in preventing thrombosis [99].

The effect of dietary Mg supplementation on BP and CV function has also been evaluated in vivo. Normotensive rats and mineralocorticoid-salt (DOCA-salt) hypertensive rats were fed for 5 weeks with a purified diet containing either a normal or Mg-supplemented diet (1.5 or 10 g/kg diet). Mg supplementation significantly lowered BP levels in hypertensive rats, but not in normotensive rats, and heart rate was not affected in either group. These findings suggested that the lowering effect of Mg supplementation on BP in hypertensive rats may be related to a vascular effect of Mg that reduces vascular tone [100]. To further test the effect of its supplementation on the development of hypertension in spontaneously hypertensive rats (SHR), Touyz et al. designed an in vivo study. Ten-week-old SHR and control Wistar-Kyoto rats (WKY) were divided into four groups which were fed for 17 weeks: WKY, WKY with Mg supplementation (650 mg/L), SHR, and SHR with Mg supplementation (650 mg/L). From 13 weeks of age, BP was significantly elevated in SHR compared with age-matched WKY. BP was significantly reduced in SHR after 10 weeks of Mg supplementation. From 18 weeks of age, serum and intracellular [Mg^2+^] levels were significantly lower in SHR than in WKY. Mg supplementation was able to normalize intracellular [Mg^2+^] and [Ca^2+^] in SHR. Overall, these results showed that mid-term Mg supplementation significantly attenuates, but does not prevent, the development of hypertension in SHR [101]. Importantly, Blache et al. tested the long-term effect of Mg-D. Rats were fed for 22 months with moderately deficient (150 mg/kg), standard (800 mg/kg), or supplemented (3200 mg/kg) Mg diets. Compared to the standard and supplemented diets, Mg-D enriched diet significantly increased BP, plasma IL-6, fibrinogen, and erythrocyte lysophosphatidylcholine. Thus, Mg-D induced a chronic impairment of redox status associated with inflammation, which could significantly contribute to increased oxidized lipids and promote hypertension and vascular disorders with aging. Extrapolating these results to the human situation and considering that Mg-D has been reported to be remarkably common—particularly among elderly individuals—Mg supplementation may be useful as an adjuvant therapy in preventing CVD [77].

### 5.5. Cardiovascular Disease Events: Arrhythmia and Acute Myocardial Infarction

Probably the most widely accepted and practiced use of Mg in CV medicine is for the prevention and/or treatment of cardiac arrhythmias [31]. Anti-arrhythmogenic properties of Mg may involve changes in the activity of some ionic channels, such as Ca^2+^ and K^+^ [102]. Both extracellular and cytosolic Mg^2+^ has significant effects on cardiac ion channels, which in turn may have important consequences on the duration of action potential, cell excitability and contractility [102]. Mg^2+^ exerts its antiarrhythmic effect via modulation of myocardial excitability. However, very few studies have evaluated the effect of Mg^2+^ on cardiac voltage-dependent Na^+^ channels. Using inside-out patches from guinea pig ventricular myocytes to measure currents through single cardiac Na^+^ channels, Mubagwa et al. [103] showed that [Mg^2+^] had no effect on inward currents but decreased the outward current amplitude in a concentration and voltage-dependent manner. This suggested that Mg^2+^ primarily exerts only an open channel blocking effect, with little or no direct allosteric modulatory action on the voltage-dependent Na^+^ channels.

Data coming from autopsies indicated that patients who had died from IHD had reduced Mg^2+^ levels in myocardium and muscle compared with those who had died from non-cardiac causes. It was observed that during myocardial ischemia, total intracellular Mg^2+^ decreases while free intracellular Mg^2+^ increases [104]. In addition, ischemia per se leads to intracellular Ca^2+^ overload, which compromises myocardial function. It was speculated that Mg^2+^ administration reduces Ca^2+^ overload as a result of the competence between these two elements for the same binding sites. In addition, the effects of Mg^2+^ on vascular tone, its ability to improve endothelial dependent vasodilation, its anticoagulant properties, possibly through improvement of NO release [105], may all exert a beneficial effect in acute myocardial infarction. In accordance with these findings, investigators started to study Mg^2+^ replacement as an adjunctive pharmacotherapy in the context of acute myocardial infarction.

### 5.6. A Focus on Magnesium Receptors

Research in the mechanisms of control of vascular Mg^2+^ homeostasis revealed two cation channels of the transient receptor potential melastatin (TRPM), TRPM6 and TRPM7, as important Mg^2+^ transporters [105]. They are differentially expressed, with TRPM6 being primarily found in epithelial cells and TRPM7 occurring ubiquitously. Vascular TRPM7 is modulated by vasoactive agents, pressure, stretch, and osmotic changes and may be a novel mechanotransducer. In addition to its Mg^2+^ transporter function, TRPM7 has been implicated as a signaling kinase involved in VSMC growth, apoptosis, adhesion, contraction, important processes involved in vascular remodeling associated with hypertension and other vascular diseases [105]. Overall, TRPM7 has been shown to play an essential role in maintaining cellular Mg^2+^ homeostasis. However, more research is needed to clarify the exact mechanisms of Mg^2+^ regulation in the CV system and the implications of abnormal transmembrane Mg^2+^ transport in the pathogenesis of vascular diseases [106]. In fact, a recent randomized, double-blind clinical trial conducted in pre-hypertensive subjects compared the effect of oral Mg supplementation versus placebo treatment for four month in the expression of different Mg^2+^ transporters in leukocytes. Rodríguez-Ramírez and collaborators reported a significant increase in the TRPM6 mRNA relative expression in leukocytes from pre-hypertensive individuals following the oral Mg supplementation compared to placebo group. Conversely, non-significant differences were reported regarding TRPM7 and solute carrier family 41 member 1 (SLC41A1) mRNA relative expression [107].

Overall, Mg is an essential microelement critical in several biological processes. Its specific role on the CV system has been widely investigated. However, further in vitro and in vivo studies are needed to explore other potential molecular targets and pathways putatively modulated and influenced by dietary and circulating Mg^2+^ levels.

## 6. Final Conclusions

Taken together, current evidence from epidemiological studies shows that higher Mg intake, either dietary or via supplementation, is associated with a protection against major CV risk factors, including MetS, T2D and hypertension/BP, as well as against stroke and total CVDs. Nevertheless, further prospective studies and RCTs are warranted to elucidate the relation between Mg intake and MetS individual components, endothelial dysfunction, lipid profile and obesity—of which current scientific knowledge remains very scarce—and HF, CHD and CVD death in different populations. Available evidence on circulating Mg and CVDs shows that greater circulating [Mg^2+^] is also associated with lower risk of CVDs, mainly IHD and CHD, yet further insights are needed to clarify the less consistent results with other CVDs and CV death. Because Mg plays a crucial role in a wide range of biological pathways and outcomes, it is not surprising that alterations in Mg homeostasis may influence different disease status.

Importantly, the fact that Mg intake is determined using indirect methods in epidemiological studies, such as food frequency questionnaires, makes it difficult to separate the observed associations from those of other microelements that may also positively contribute to cardiometabolic health. Thus, a residual effect from the intake of other dietary microelements cannot be discarded despite the efforts for controlling this in multivariate models.

Traditionally, supplement formulations from organic Mg (aspartate, citrate, lactate and chloride) have been considered to be more bioavailable than those with inorganic Mg (oxide and sulfate), as reported by a number of studies [108]. However, this topic is currently under debate given that other studies have found no differences between these formulations, and several factors have been shown to play a role in the complex process of Mg absorption and utilization [109].

The inverse association between Mg intake and IR, hyperglycemia, dyslipidemia, hypertension, and markers of inflammation may justify the protective effect of dietary Mg on CVD. Overall, the current evidence supports the importance of adequate dietary magnesium for lowering CVD risk. In addition, these findings support the importance to increase the consumption of magnesium-rich foods, including fruits, vegetables, legumes, nuts and whole grains for the prevention of chronic diseases.

## Figures and Tables

**Table 1:** List of foods and food groups from plant and animal origin with their corresponding magnesium content (mg/100 g edible food).

Food from Plant Origin		Food from Animal Origin	
**Nuts and Seeds**	Mg mg/100 g	**Dairy and Eggs**	Mg mg/100 g
Pumpkin seeds, dried	592	Parmesan cheese	44
Flaxseed	392	Feta cheese	19
Sesame seeds, roasted	356	Whole-fat milk	13
Almonds, raw	270	Plain whole-fat yogurt	12
Cashew nuts, roasted	260	Whole fresh egg	12
Walnuts	158		
Pistachio nuts, roasted	109		
**Legumes**		**Fish and seafood**	
Peanuts, roasted	178	Cod, cooked	133
Soybeans, cooked	86	Salmon, cooked	122
Chickpeas, cooked	48	Canned anchovies	69
Kidney beans, cooked	45	Shrimps, cooked	37
Lentils, cooked	36		
**Vegetables and fruits**		**Meat and meat products**	
Sun-dried tomatoes	194	Chicken breast, cooked	34
Spinach, cooked	87	Turkey, cooked	32
Kale, cooked	57	Veal, cooked	34
Dates	54	Rabbit, cooked	21
Fresh parsley	50		
Baked potatoes with skin	43		
**Whole grains**			
Buckwheat flour	251		
Amaranth grain	248		
Quinoa grain	197		
Oats	177		
Spelt	136		
Barley	133		

Data obtained from the US Department of Agriculture (USDA), Nutrient Database for Standard Reference, Release 28 [10].

## Abbreviations

ARIC	Atherosclerosis Risk in Communities
ATP	adenosine triphosphate
BMI	body mass index
BP	blood pressure
Ca^2+^	calcium
CHD	coronary heart disease
CV	cardiovascular
CVD	cardiovascular disease
DNA	deoxyribonucleic acid
GLUT 4	glucose transporter protein activity 4
HDL	high-density lipoprotein
HF	heart failure
HR	hazard ratio
HOMA-IR	homeostatic model assessment of insulin resistance
IHD	ischemic heart disease
IL	interleukin
IR	insulin resistance
K+	potassium
LDL	low-density lipoprotein
MetS	metabolic syndrome
Mg	magnesium
Mg-D	Mg-deficiency
[Mg^2+^]	magnesium concentrations
Mg^2+^	intracellular magnesium
Na+	sodium
NO	nitric oxide
O2	oxygen
T2D	type 2 diabetes
TGRLP	triglyceride-rich lipoproteins
TRPM	transient receptor potential melastatin
RCT	randomized control trial
RDA	recommended dietary allowance
RR	Relative Risk
sICAM-1	soluble intercellular adhesion molecule 1
SLC41A1	solute carrier family 41 member 1
SHR	spontaneously hypertensive rats
UVEC	umbilical vein endothelial cells
VCAM	vascular cell adhesion protein 1
VSMC	vascular smooth muscle cells
WKY	Wistar-Kyoto rats
